# Epstein-Barr virus encoded miR-BART11 promotes inflammation-induced carcinogenesis by targeting FOXP1

**DOI:** 10.18632/oncotarget.9170

**Published:** 2016-05-04

**Authors:** Yali Song, Xiaoling Li, Zhaoyang Zeng, Qiao Li, Zhaojian Gong, Qianjin Liao, Xiayu Li, Pan Chen, Bo Xiang, Wenling Zhang, Fang Xiong, Yanhong Zhou, Ming Zhou, Jian Ma, Yong Li, Xiang Chen, Guiyuan Li, Wei Xiong

**Affiliations:** ^1^ The Key Laboratory of Carcinogenesis and Cancer Invasion of The Chinese Ministry of Education, Xiangya Hospital, Central South University, Changsha, Hunan, China; ^2^ The Key Laboratory of Carcinogenesis of The Chinese Ministry of Health and Cancer Research Institute, Central South University, Changsha, Hunan, China; ^3^ Hunan Key Laboratory of Nonresolving Inflammation and Cancer, Disease Genome Research Center, The Third Xiangya Hospital, Central South University, Changsha, Hunan, China; ^4^ Hunan Key Laboratory of Translational Radiation Oncology, Hunan Cancer Hospital and The Affiliated Cancer Hospital of Xiangya School of Medicine, Central South University, Changsha, Hunan, China; ^5^ Department of Cancer Biology, Lerner Research Institute, Cleveland Clinic, Cleveland, Ohio, USA

**Keywords:** nasopharyngeal carcinoma, gastric cancer, Epstein-Barr virus, EBV-miR-BART11, FOXP1

## Abstract

Epstein-Barr virus (EBV) infection and chronic inflammation are closely associated with the development and progression of nasopharyngeal carcinoma (NPC) and gastric cancer (GC), and the infiltration of inflammatory cells, including tumor-associated macrophages (TAMs), is often observed in these cancers. EBV encodes 44 mature micro RNAs (miRNAs), but the roles of only a few EBV-encoded miRNA targets are known in cancer development, and here, our aim was to elucidate the effects of EBV-miR-BART11 on FOXP1 expression, and potential involvement in inflammation-induced carcinogenesis. We constructed an EBV miRNA-dependent gene regulatory network and predicted that EBV-miR-BART11 is able to target forkhead box P1 (FOXP1), a key molecule involved in monocyte to macrophage differentiation. Here, using luciferase reporter assay, we confirmed that EBV-miR-BART11 directly targets the 3′-untranslated region of *FOXP1* gene, inhibits FOXP1 induction of TAM differentiation, and the secretion of inflammatory cytokines into the tumor microenvironment, inducing the proliferation of NPC and GC cells. FOXP1 overexpression hindered monocyte differentiation and inhibited NPC and GC cells growth. Our results demonstrated that EBV-miR-BART11 plays a crucial role in the promotion of inflammation-induced NPC and GC carcinogenesis by inhibiting FOXP1 tumor-suppressive effects. We showed a novel EBV-dependent mechanism that may induce the carcinogenesis of NPC and GC, which may help define new potential biomarkers and targets for NPC and GC diagnosis and treatment.

## INTRODUCTION

Chronic inflammation is an important mediator of nasopharyngeal carcinoma (NPC) [1–3] and gastric cancer (GC) [4]. A large number of inflammatory cells, including tumor-associated macrophages (TAMs), are found in NPC and GC biopsies. TAM infiltration is tightly associated with poor prognosis in NPC [5, 6]. TAMs were shown, together with tumor-derived FasL, to serve as a barrier against the infiltration of CD8+ T cells into GC [7, 8]. Furthermore, our previous studies demonstrated that TAM-derived inflammatory factors, such as IL-6, stimulate NPC cell proliferation [9, 10].

Epstein-Barr virus (EBV) infection is closely associated with the development and progression of NPC and GC [11–13]. EBV encodes 44 mature microRNAs (miRNAs), divided into two clusters, BHRFs and BARTs [14–16]. BART miRNAs were shown to affect the malignant phenotype of some lymphomas [17], GC [18], and NPC [19–22], including viral latency [23–25], immune escape [26], cell proliferation [27], cell apoptosis [28, 29], cell cycle regulation, and cancer metabolism [27–30]. These findings suggest that EBV miRNAs may exert a variety of important regulatory functions in EBV-mediated tumorigenesis and cancer progression. Nevertheless, the functions of most EBV-encoded miRNAs remain to be elucidated.

We previously profiled all 44 EBV-encoded mature miRNAs in NPC biopsies and non-cancerous nasopharyngeal tissues, and found that EBV miRNAs located in the BART region were highly expressed in NPC biopsies [31–33]. Additionally, we constructed an EBV miRNA-dependent gene regulatory network and determined that both the 3′- and 5′-arms of the mature EBV-miR-BART11 hairpin precursor may specifically target forkhead box P1 (*FOXP1*) mRNA. FOXP1 has been reported to be involved in monocyte differentiation to macrophage, where its downregulation represents a key molecular event [34, 35]. However, the biological functions of both EBV-miR-BART11 and FOXP1 in EBV-associated carcinogenesis have not been defined yet. We hypothesized that EBV may participate in TAM differentiation and promote inflammation-induced EBV-associated carcinogenesis via EBV-miRNA-BART11 expression and subsequent FOXP1 downregulation.

## RESULTS

### FOXP1 is a target of EBV-miR-BART11

**Figure 1:**
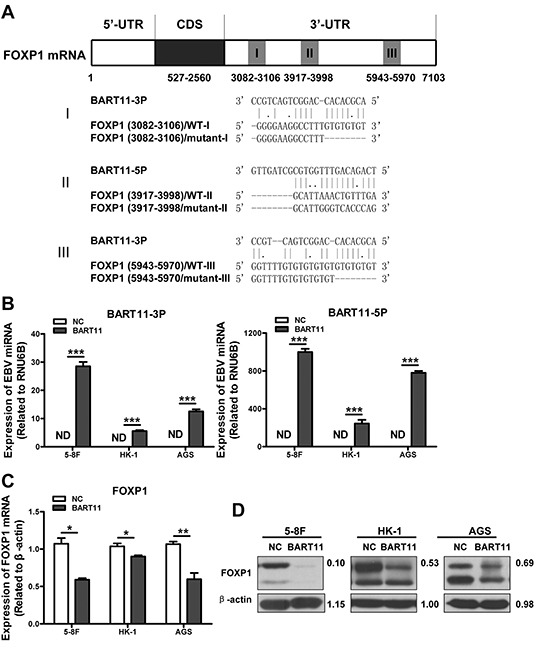

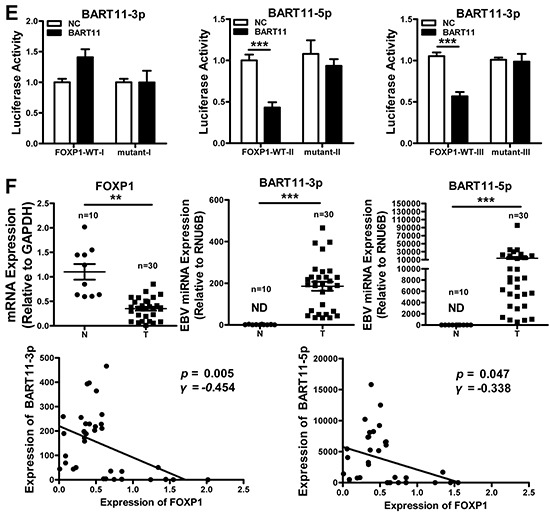
FOXP1 is a direct target of EBV-miR-BART11 **A.** Three binding sites of EBV-miR-BART11-3p and EBV-miR-BART11-5p were predicted in the *FOXP1* 3′-UTR, including 3082 bp-3106 bp (I), 3917 bp-3998 bp (II), and 5943 bp-5970 bp (III). Wild-type (FOXP1-WT) and mutant (FOXP1-mutant) sequences were used to validate these predictions. **B.** The expression of exogenous EBV-miR-BART11-3p (left) and EBV-miR-BART11-5p (right) was detected by qRT-PCR. FOXP1 **C.** mRNA and **D.** protein expression levels in 5-8F, HK-1, and AGS cells after EBV-miR-BART11 treatment. β-actin served as loading control. **E.** Luciferase reporter assay, using reporter vectors containing either wild-type (FOXP1-WT) or mutant (FOXP1-mutant) *FOXP1* 3′-UTR, and EBV-miR-BART11 or non-targeting control, was performed in order to identify the direct binding of EBV-miR-BART11 to the *FOXP1* 3′-UTR in 5-8F cells. **F.** EBV-miR-BART11 and FOXP1 expression levels in NPC and control specimens were detected by qRT-PCR. N, non-tumor nasopharyngeal epithelium (n = 10); T, NPC (n = 30). Representative images or data expressed as mean ± SD of the measurements obtained in three separate experiments are presented (ND: not detected; *p < 0.05; **p < 0.01;***p < 0.001).

Bioinformatic analysis identified three putative EBV-miR-BART11 binding sites (two for EBV-miR-BART11-3p and one for EBV-miR-BART11-5p) in the *FOXP1* 3′-untranslated region (UTR) (Figure 1A). To determine the effect of EBV-miR-BART11 on FOXP1 expression, EBV-miR-BART11 precursor vector expressing both mature EBV-miR-BART11-3p and EBV-miR-BART11-5p was constructed and transfected into three different EBV-negative cancer cell lines (5-8F, HK-1, and AGS). The expression of mature EBV-miR-BART11-3p and EBV-miR-BART11-5p was measured using qRT-PCR (Figure 1B). These results demonstrated that the EBV-miR-BART11 precursor vector can successfully express mature EBV-miR-BART11-3p and EBV-miR-BART11-5p in EBV-negative cancer cell lines. Further analysis revealed that EBV-miR-BART11 significantly inhibited FOXP1 expression at the mRNA and protein levels when compared with empty vector controls in 5-8F, HK-1, and AGS cells (Figure 1C–1D).

To elucidate if FOXP1 is a direct target of EBV-miR-BART11-3p and EBV-miR-BART11-5p, three pairs of luciferase reporter vectors containing either wild-type (WT-I, WT-II, and WT-III) EBV-miR-BART11 binding or mutant sequences of the *FOXP1* 3′-UTR were co-transfected with the EBV-miR-BART11 precursor expression vector in 5-8F cells. EBV-miR-BART11 significantly attenuated the luciferase activity of FOXP1-WT vectors II and III, but exhibited no effects on the FOXP1-WT-I vector or the FOXP1-mutant vectors (Figure 1E). These results suggested that EBV-miR-BART11 is able to inhibit FOXP1 expression by targeting the binding sites II and III in the *FOXP1* 3′-UTR.

To explore the relationship between EBV-miR-BART11 and FOXP1, EBV-miR-BART11-(3p and 5p) and *FOXP1* mRNA expression was assessed in 30 NPC biopsies and 10 non-tumor nasopharyngeal epithelial tissues. As expected, EBV-miR-BART11-3p and EBV-miR-BART11-5p expression levels were significantly higher in NPC samples than in normal nasopharyngeal epithelial samples, and these levels were negatively correlated with *FOXP1* expression (*p* < 0.05, Figure 1F).

### EBV-miR-BART11 promotes monocyte differentiation by attenuating FOXP1 expression

**Figure 2:**
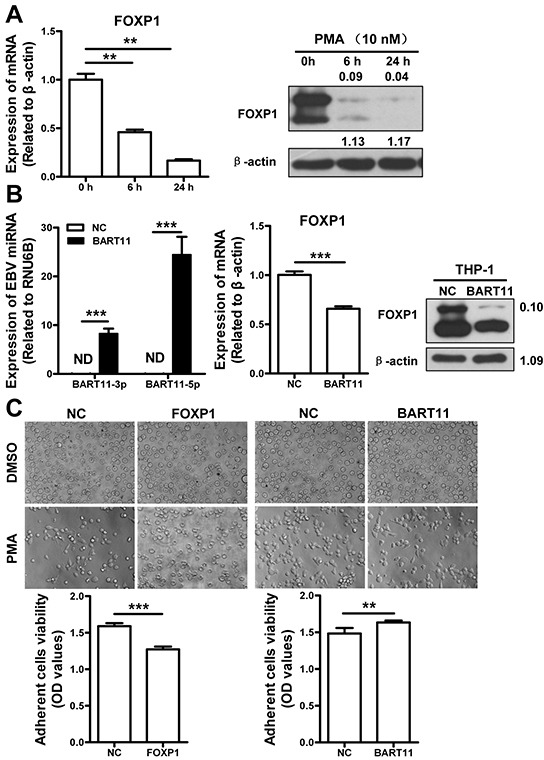
EBV-miR-BART11 promotes monocyte differentiation of THP-1 cells by attenuating FOXP1 expression **A.** FOXP1 expression at mRNA (left) and protein (right) levels, during the PMA-induced differentiation of monocytic THP-1 cells. **B.** The expression of EBV-miR-BART11 (left) and FOXP1 (middle, mRNA; right, protein) was examined by qRT-PCR and western blotting, respectively, in THP-1 cells infected with lentivirus encoding EBV-miR-BART11. **C.** The effects of EBV-miR-BART11 and FOXP1 on monocyte differentiation. Morphological changes were monitored in PMA-induced THP-1 cells following FOXP1 overexpression vector or EBV-miR-BART11 precursor vector transfection. THP-1 cell differentiation was determined by the viability of adherent cells, using MTT assay. Data represent mean ± SD of OD values obtained in three separate experiments (ND: not detected; **p < 0.01; ***p < 0.001).

In order to define the relationship between EBV-miR-BART11 and FOXP1 in monocyte to macrophage differentiation further, we monitored temporal FOXP1 expression in THP-1 monocytes subjected to PMA-induced macrophage differentiation. This revealed that FOXP1 is dramatically downregulated during monocyte to macrophage transformation (Figure 2A), which is consistent with the previous reports [34, 35]. Therefore, we hypothesized that EBV-miR-BART11 may stimulate monocyte differentiation. To test this theory, THP-1 cells were infected with lentivirus encoding FOXP1 or EBV-miR-BART11, and treated with PMA to induce differentiation. The results revealed that EBV-miR-BART11 downregulated both FOXP1 mRNA and protein expression (Figure 2B). In addition, we found that FOXP1 expression hindered PMA-induced THP-1 differentiation, whereas EBV-miR-BART11 overexpression was shown to induce this process, compared with the negative control (Figure 2C).

### EBV-miR-BART11-expressing macrophages are hyperresponsive to LPS

**Figure 3:**
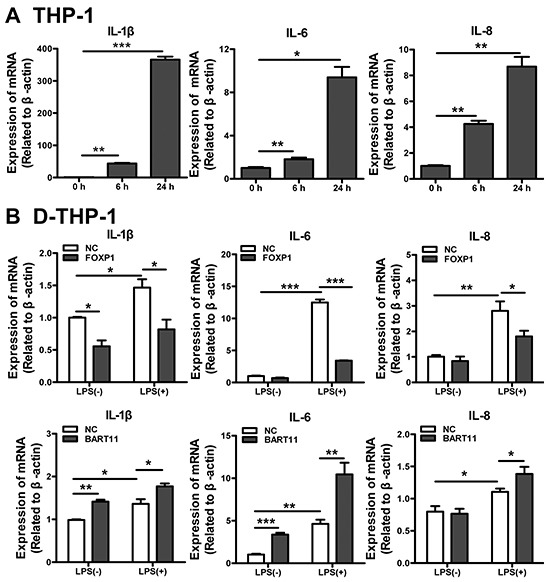
EBV-miR-BART11-transfected macrophages are hyperresponsive to LPS **A.** Pro-inflammatory cytokine (IL-1β, IL-6, and IL-8) expression in PMA-treated THP-1 cells. **B.** Differentiated THP-1 (D-THP-1) cells, stimulated with LPS in the presence of exogenous FOXP1 overexpression (upper panel) or EBV-miR-BART11 (lower panel). Data are expressed as mean ± SD of the results obtained from each group of cells in three separate experiments (*p < 0.05; **p < 0.01; ***p < 0.001).

Macrophages play a key role in chronic inflammation and can trigger a pro-inflammatory response by secreting inflammatory factors [36, 37]. The expression of several prototypical pro-inflammatory cytokines (IL-1β, IL-6, and IL-8) markedly increased in PMA-induced THP-1 monocytes (D-THP-1), compared with the untreated controls (Figure 3A). In order to investigate the effects of FOXP1 and EBV-miR-BART11 on LPS-induced pro-inflammatory cytokine production in D-THP-1 cells, the investigated cells were infected with lentivirus encoding FOXP1 or EBV-miR-BART11. Notably, IL-1β, IL-6, and IL-8 mRNA expression in FOXP1-overexpressing cells was significantly lower than in controls. Elevated cytokine levels were detected in both the control and FOXP1-overexpressing cells stimulated with LPS (1 μg/mL) for 24 h; however, the levels in FOXP1-overexpressing cells were shown to be significantly lower than those in controls. Together, these data demonstrated that FOXP1 attenuates spontaneous and LPS-stimulated IL-1β, IL-6, and IL-8 expression in D-THP-1 cells, whereas EBV-miR-BART11 has the opposite effect (Figure 3B).

### EBV-miR-BART11 enhances the local inflammatory response in carcinoma

**Figure 4:**
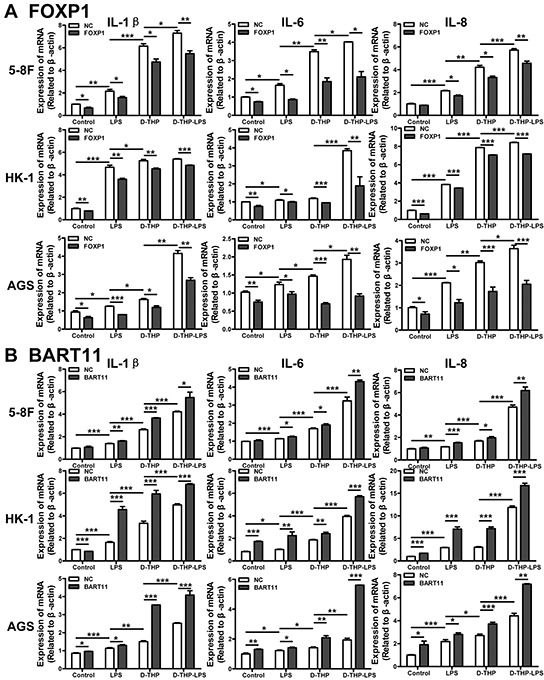
Effect of FOXP1 and EBV-miR-BART11 expression on the local inflammatory response in epithelial cells The expression of inflammatory factors (IL-1β, IL-6, and IL-8), measured in 5-8F and HK-1 NPC and AGS GC epithelial cells transfected with FOXP1 overexpression vector **A.** or EBV-miR-BART11 precursor vector **B.** and treated with LPS or conditioned media collected from the untreated differentiated-THP-1 cells (D-THP) or LPS-treated D-THP-1 cells (D-THP-LPS). Data are represented as mean ± SD of three independent experiments. *p < 0.05; **p < 0.01; ***p < 0.001.

TAMs are a major component of the immune infiltrates present in the NPC microenvironment, where they produce cytokines, growth factors, and angiogenic inducers that amplify the inflammatory response and promote tumor survival and growth [38]. The respective contributions of FOXP1 and EBV-miR-BART11 to this process in nasopharyngeal epithelial cells remain unclear. Therefore, we examined IL-1β, IL-6, and IL-8 production in EBV-negative cell lines (5-8F, HK-1, and AGS) following LPS treatment. Naïve EBV-negative cells were treated with conditioned media collected from LPS-treated D-THP-1 cells, in order to mimic the tumor microenvironment. IL-1β, IL-6, and IL-8 mRNA expression increased in EBV-negative epithelial cells following LPS treatment, which was potentiated after culturing in conditioned media. Consistent with our earlier findings [6], exogenous expression of FOXP1 or EBV-miR-BART11 was shown to significantly reduce or potentiate the local inflammatory response, respectively (Figure 4A–4B).

### EBV-miR-BART11 accelerates cell proliferation by attenuating FOXP1 expression

**Figure 5:**
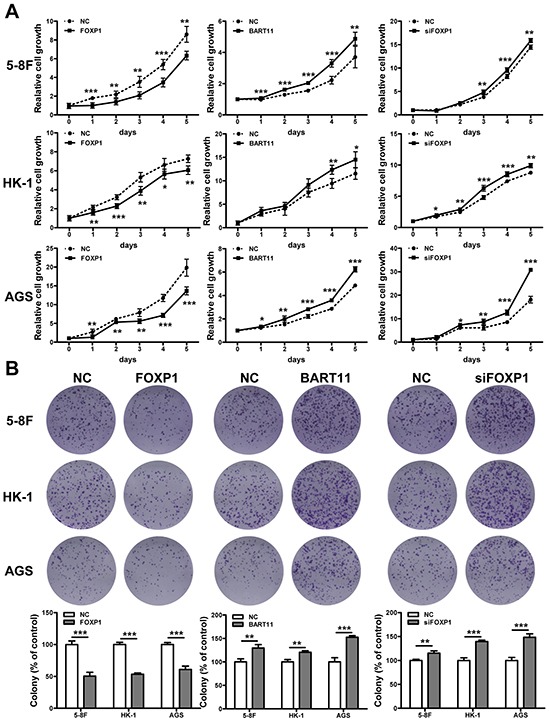

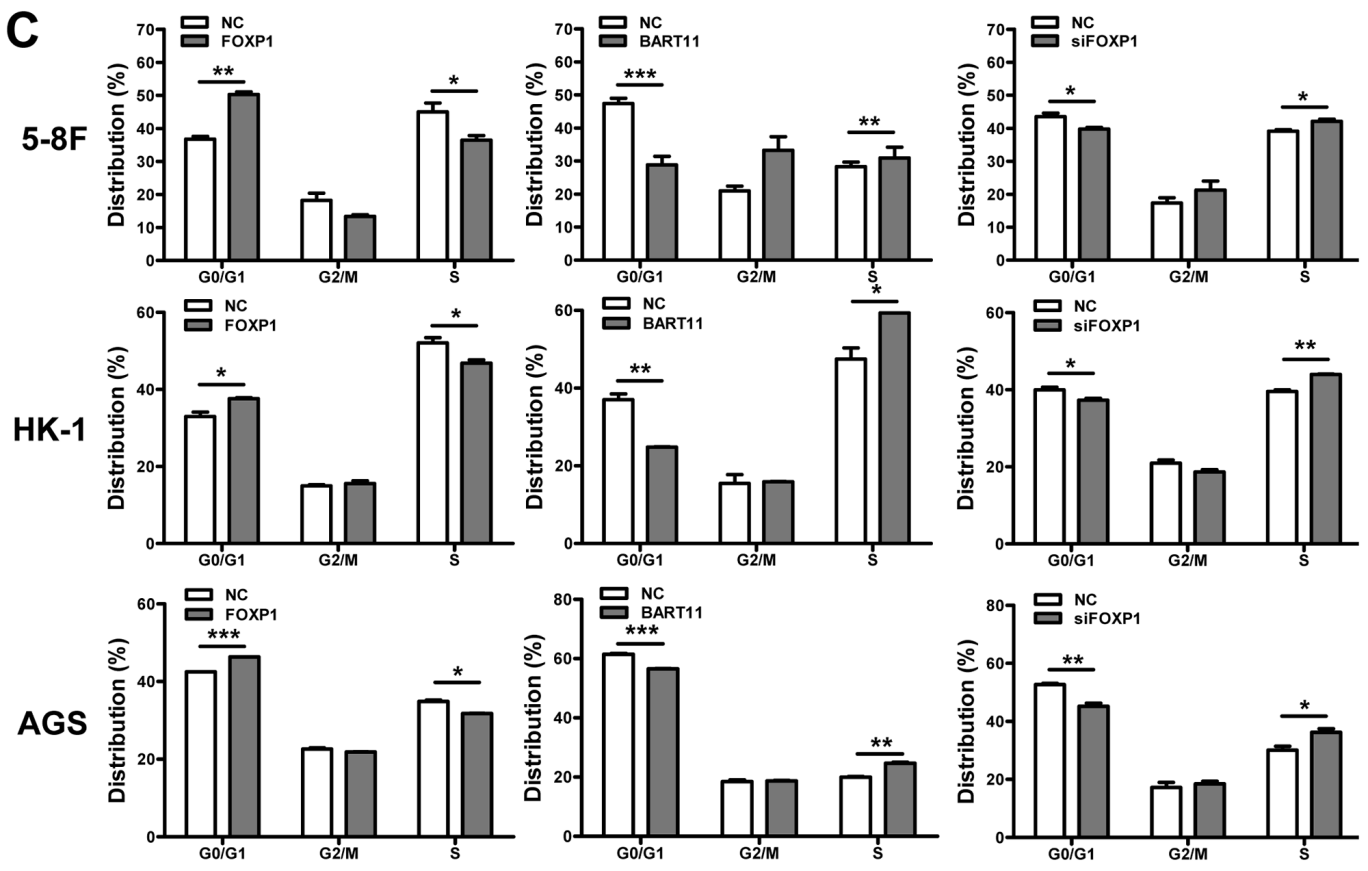
Effect of EBV-miR-BART11 and FOXP1 on cancer cell proliferation The effect of FOXP1 or EBV-miR-BART11 overexpression, or FOXP1 siRNA knockdown (siFOXP1) on cell proliferation was investigated by the **A.** MTT assay and **B.** colony formation assay in 5-8F, HK-1, and AGS cells. **C.** Cell cycle analysis of cell cycle progression by flow cytometric analysis. All data were normalized to the vector-only control cell proliferation rate, and the results are expressed as mean ± SD of the results obtained in three independent experiments (*p < 0.05; **p < 0.01; ***p < 0.001).

Cytokines stimulated by the local inflammatory response promote epithelial cell carcinogenesis through the induction of cell proliferation [5, 9]. In order to understand the roles of EBV-miR-BART11 and FOXP1 in epithelial cell proliferation, EBV-negative cell lines were transfected with EBV-miR-BART11, FOXP1, or FOXP1 siRNAs (siFOXP1), and cell proliferation was examined by the MTT assay. siFOXP1 was demonstrated to induce FOXP1 knockdown specifically, at both mRNA and protein levels (Supplementary Figure S1). Both EBV-miR-BART11 overexpression and FOXP1 silencing enhanced cell proliferation, while FOXP1 overexpression had the opposite result (Figure 5A). These findings were validated using the colony formation assay as well, which showed that FOXP1 expression significantly reduced colony forming potential, in terms of both colony number and size. Similarly, EBV-miR-BART11 and siFOXP1 exhibited the opposite effects on colony formation potential (Figure 5B). Furthermore, flow cytometric analysis revealed that FOXP1 overexpression results in a higher percentage of cell accumulation in G0/G1 phase, compared with the non-transfected controls (Figure 5C).

### EBV-miR-BART11 promotes inflammation-induced cell proliferation by suppressing FOXP1 expression

**Figure 6:**
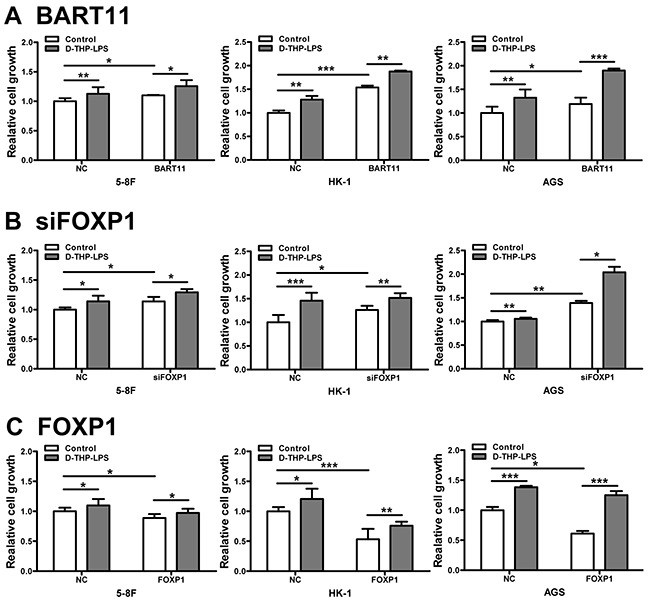
Effect of EBV-miR-BART11 and FOXP1 on inflammation-induced cancer cell proliferation The proliferation of 5-8F, HK-1, and AGS cells transfected with EBV-miR-BART11 precursor vector **A.** FOXP1 siRNA **B.** or FOXP1 overexpression vector **C.** cultured in the conditioned media collected from LPS-treated differentiated THP-1 cells (D-THP-LPS). Data are expressed as mean ± SD of cell growth following different treatments, of three independent experiments. *p < 0.05; **p < 0.01; ***p < 0.001.

Our previous study indicated that LPS-stimulated TAMs can promote epithelial cell proliferation [5]. In order to understand the functions of EBV-miR-BART11 and FOXP1 in inflammation-induced cell proliferation, 5-8F, HK-1, and AGS cells with exogenous EBV-miR-BART11 or FOXP1 expression, or FOXP1 knockdown cells were cultured in conditioned media obtained from LPS-stimulated D-THP-1 cells. These results showed that EBV-miR-BART11 overexpression (Figure 6A) or FOXP1 knockdown (Figure 6B) can promote inflammation-induced cell proliferation, whereas FOXP1 overexpression inhibited this effect (Figure 6C).

### EBV-miR-BART11 activates NF-κB signaling through FOXP1

**Figure 7:**
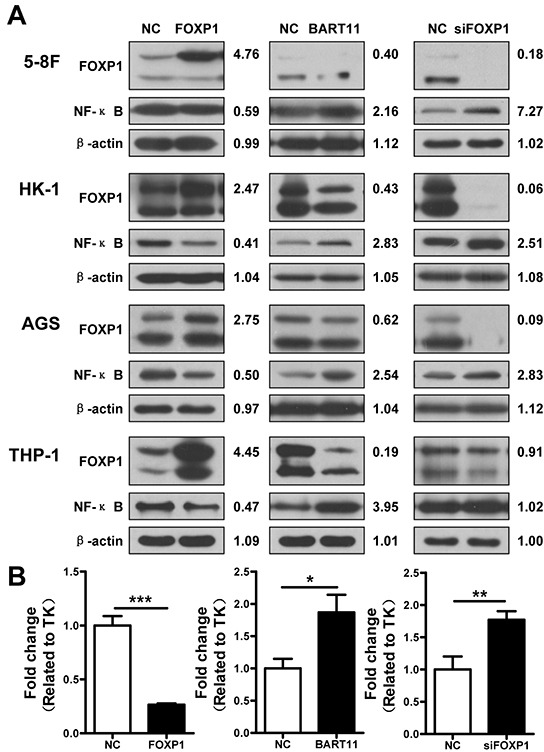
EBV-miR-BART11-mediated NF-κB activation via FOXP1 inhibition **A.** The expression of FOXP1 and NF-κB in 5-8F, HK-1, and AGS epithelial cancer cells and THP-1 monocytes, transfected with FOXP1, EBV-miR-BART11, or siFOXP1, and determined by western blotting and densitometry. **B.** NF-κB transcriptional activity, assessed by luciferase reporter assay in 5-8F cells transfected with the FOXP1 overexpression vector, EBV-miR-BART11 precursor vector, or siFOXP1. Data shown are representative images or expressed as mean ± SD of different groups of cells from three independent experiments. *p < 0.05; **p < 0.01; ***p < 0.001.

Nuclear factor κB (NF-κB) orchestrates the inflammatory response by regulating the expression of numerous genes [5, 36]. Therefore, we investigated the effects of FOXP1 and EBV-miR-BART11 on NF-κB p65 expression in THP-1, 5-8F, HK-1, and AGS cells. FOXP1 overexpression markedly decreased NF-κB p65 protein expression, while EBV-miR-BART11 overexpression or FOXP1 knockdown led to an increase in NF-κB p65 expression (Figure 7A). The analysis of NF-κB transcriptional activity using the reporter assay in 5-8F cells revealed that exogenous FOXP1 expression led to the inhibition of NF-κB transcriptional activity, while EBV-miR-BART11 or FOXP1 siRNA had the opposite effect (Figure 7B).

## DISCUSSION

Chronic, non-resolving inflammation plays an important role in the initiation of a variety of tumors, including NPC [38, 39] and GC [40–42]. While various immune cells are involved in chronic inflammation, TAMs represent the key mediators of chronic inflammation within the tumor microenvironment [37]. We previously found that TAM infiltration promotes NPC cell proliferation by the secretion of inflammatory factors, such as IL-6 [5, 9], and induce NF-κB and STAT3 transcription factor activation [39, 43], ultimately resulting in poor disease prognosis.

EBV-infected macrophages induce an inflammatory response in EBV-related human tumors [44–46], but the relationships between EBV-encoded miRNAs and TAMs in EBV-associated solid tumors have not been reported. In our previous study [2], we constructed an EBV miRNA-dependent gene regulatory network and predicted that the EBV-encoded miRNAs, BART11-3p and BART11-5p, may regulate FOXP1 expression [2]. FOXP1 belongs to the FOX transcription factor family, which includes a variety of ‘winged helix’ transcription factors that play crucial roles in immune homeostasis [47]. Several members of the FOX family, for example FOXF1, FOXP3, FOXN1, FOXO1, and FOXO3, were shown to execute diverse functions during the regulation of inflammation and adaptive immune response [48–51]. The *FOXP1* locus is located on 3p14.1 [47], a region known for its high-frequency allele imbalance [52–54], and it is a defined genetic susceptibility region [55, 56] in NPC. Generally, FOXP1 acts as a transcriptional repressor that regulates the differentiation of B [57] and T lymphocytes [58], monocytes [34, 59], and other immune cells. Therefore, we speculated that EBV may alter immune cell differentiation and the inflammatory process, in order to promote inflammation-induced carcinogenesis in EBV-associated cancers by inhibiting FOXP1 expression.

We confirmed *FOXP1* as a target of EBV-miR-BART11 in both monocytes and epithelial cells, and showed that FOXP1 downregulation was necessary for monocyte differentiation. We further investigated the effects of EBV-miR-BART11 and FOXP1 on macrophage cytokine secretion. EBV-miR-BART11 overexpression in TAMs resulted in LPS hyperresponsiveness and the increased secretion of cytokines, chemokines, and growth factors that support tumor survival, proliferation, and invasion. Taken together, our results indicate that EBV-miR-BART11 is sufficient to maintain and potentiate the inflammatory environment by enhancing macrophage differentiation and responsiveness through the downregulation of FOXP1.

Under certain conditions, tumor cells can induce their own inflammatory factor secretion, in order to intensify the local inflammatory microenvironment, resulting in further TAM and lymphocyte infiltration [60]. Therefore, we investigated the effects of EBV-miR-BART11 and FOXP1 on the local inflammatory response in epithelial cells. Our results showed that LPS stimulation significantly enhances cytokine secretion in both macrophages and epithelial cells, and that the conditioned media collected from LPS-treated macrophages is able to stimulate epithelial cells to produce an even higher level of cytokines.

FOXP1 acts as a transcriptional repressor in lymphocytes where its downregulation plays important roles in immune cell differentiation. It is also frequently downregulated in solid tumors, including breast cancer [61–63], non-small cell lung cancer [64], oral squamous cell carcinoma [65], ovarian cancer [66, 67], renal cell carcinoma [68, 69], hepatocellular carcinoma [70] and prostate cancer [71–73], which suggests that FOXP1 may act as a tumor suppressor. However, its role in EBV-associated solid tumors, such as NPC and GC, is yet to be completely understood. Our results demonstrate that tumor-suppressive effects of FOXP1 inhibit epithelial cell proliferation, which can be counteracted with EBV-miR-BART11 expression.

NF-κB transcriptional regulation plays a critical role in tumorigenic inflammation. FOXP1 and NF-κB interact through a complex regulatory mechanism involved in several biological processes that vary in a cell type-dependent manner. In lymphocytes, FOXP1 is able to inhibit directly apoptotic gene transcription and cooperate with NF-κB, promoting human B cell survival, while FOXP1 expression is dependent on NF-κB transcriptional activity and plays a synergistic role in NF-κB self-activation [74]. NF-κB activation in malignant B cells can increase FOXP1 expression [75], but some results indicate that full-length FOXP1 may function as a NF-κB transcriptional repressor in follicular lymphoma [76, 77]. Conversely, NF-κB has also been shown to downregulate FOXP1 in monocyte differentiation and macrophage function [34], and therefore, downregulation of the NF-κB signaling pathway is an effective mechanism of FOXP1-mediated inhibition of monocyte differentiation and macrophage function. The regulatory relationships between EBV-miR-BART11, FOXP1, NF-κB, and their downstream signaling pathways, and their detailed biological functions, require further investigations.

In conclusion, we revealed EBV-miR-BART11 plays an important role in the inflammatory microenvironment and inflammation-induced carcinogenesis in EBV-associated cancers through the direct inhibition of FOXP1 and NF-κB activation. Collectively, these data demonstrate that EBV-miR-BART11 or FOXP1 may serve as potential diagnostic or prognostic markers in NPC or GC, and may represent important targets for EBV-related cancer immunotherapy.

## MATERIALS AND METHODS

### Cell lines and reagents

Two EBV-negative human NPC cell lines (5-8F and HK-1), AGS GC cells, and THP-1 monocytes were grown in RPMI-1640 medium supplemented with 10% fetal calf serum (FCS) at 37°C in the atmosphere with 5% CO_2_. THP-1 cells were treated with 10 nM phorbol 12-myristate 13-acetate (PMA, Sigma, St Louis, MO, USA) to induce monocyte differentiation. Lipopolysaccharide (LPS) from *Escherichia coli* 0111:B4 was obtained from Sigma (10 ng/mL). 293T cells used to produce the lentiviral stock were grown in Dulbecco's modified Eagle's medium (DMEM) supplemented with 10% FCS, at 37°C in the atmosphere containing 5% CO_2_.

### siRNAs, EBV-miR-BART11 and FOXP1 overexpression vectors, and cell transfection

FOXP1-specific siRNAs and their corresponding controls were synthesized by Ruibo (Guangzhou, China) (Supplementary Table S1). EBV-miR-BART11 precursor sequence was synthesized (Invitrogen, Shanghai, China) and cloned into the pSUPER.neo/GFP vector (Invitrogen, Shanghai, China). The *FOXP1* full-length coding sequence (CDS, NM_032682) was amplified and cloned into the pIRESneo3 vector (Invitrogen). Cell transfection was performed in 70-80% confluent cells using Lipofectamine 3000 (Invitrogen) according to the manufacturer's protocol.

### Lentiviral vectors and THP-1 cell infection

Lentiviral vector construction was constructed using the ViraPower™ Lentiviral Expression System (Invitrogen). First, EBV-miR-BART11 precursor or full-length *FOXP1* CDS was subcloned into the pLenti6/V5-D-TOPO vector. pLenti6/V5-D-TOPO/BART11 or pLenti6/V5-D-TOPO/FOXP1 vectors and ViraPower Packaging Mix were co-transfected using a gene carrier kit (Epoch-Biolabs, Missouri City, TX, USA) into 293T cells to produce a lentiviral stock, and 48 h after transfection, virus-containing supernatant was collected and used to infect THP-1 at a ratio of 1:1 with fresh medium, as described previously [78].

### Quantitative real time polymerase chain reaction (qRT-PCR)

Total RNA was harvested using the TRIzol Extraction Kit (Invitrogen) and cDNA samples were prepared using a QuantiTect Reverse Transcription Kit (Qiagen, Hilden, Germany). Stem-loop real-time qRT-PCR for mature miRNAs was done with the Qiagen QuantiTect SYBR Green PCR Kits (Qiagen), using RNU6B (U6) as an international control. qRT-PCR for mRNA expression was performed using the SYBR Premix Ex Taq II kit (Takara, Dalian, China) according to the manufacturer instructions with *β-actin* or *GAPDH* as the internal controls. The primers used are shown in Supplementary Table S1.

### Western blotting

Whole cell lysates were extracted using RIPA lysis buffer (50 mM Tris-HCl pH 7.4, 250 mM NaCl, 0.1% SDS, 0.5% NP-40, 2 mM DTT, 1 mg/mL protease inhibitors). Protein concentration was quantified using a BCA protein assay kit (Pierce, Grand Island, NY). Afterward, 50 μg of protein was resolved by 10% sodium dodecyl sulfate-polyacrylamide gel electrophoresis (SDS-PAGE) and electroblotted onto a PVDF membrane (Millipore, Billerica, MA, USA). Immunodetection using antibodies against FOXP1 (Cell Signaling, Danvers, MA), NF-κB p65 (Abcam, MA, USA), or β-actin (Millipore) was done for 1 h at room temperature or overnight at 4°C. Antibody-antigen complexes were detected using the enhanced chemiluminescence (ECL) system (Amersham Bioscience, Piscataway, NJ, USA). β-actin served as a loading control.

### Methylthiazol tetrazolium assay (MTT) and colony formation assay

Cell proliferation was measured using an MTT (Millipore) assay. After transfection or treatment, 800 cells per well were seeded in 96-well plates, and viability was assessed in five replicates at designed times. The formation of colored formazan dye was measured at 490 nm on a spectrophotometric plate reader.

For the clonogenic assays, 1000 cells were plated in six-well plates at 24 h post-transfection and incubated for 14 days. The number of colonies was counted with Image-Pro Plus (Syngene, Frederick, MD, USA) and normalized to the control group. The experiments were performed at least three times.

### Flow cytometry

Cell cycle analysis was performed using flow cytometry with propidium iodide (PI) staining (5 μg/mL) 48 h post-transfection. Events were recorded from samples using a FACSCalibur Flow Cytometer (BD Biosciences, New Jersey, USA) equipped with Cell Quest 3.3 software and analyzed using ModFit software (Verity Software House, Topsham, ME).

### Luciferase reporter assay

5-8F cells were co-transfected with synthetic EBV-miR-BART11 and luciferase reporter vectors (FOXP1-WT or FOXP1-mutant) along with pRL-TK *Renilla* luciferase vector (Promega, Madison, WI). For NF-κB reporter assay, 5-8F cells were transiently co-transfected with the pNF-κB-Luc construct (Promega), pRL-TK vector, and EBV-miR-BART11 or the FOXP1 expression vector or siFOXP1. Luciferase activity was determined by the Dual Luciferase Assay Kit (Promega) 48 h after transfection [79–81]. Transfections were performed in duplicates and repeated in three independent experiments.

### Statistical analysis

Statistical analyses were performed using SPSS 13.0 (SPSS, Chicago, IL) and Graph Pad Prism 5 (GraphPad, La Jolla, CA). Data are presented as mean ± standard deviation (SD). Comparisons between two groups were performed using Student's t-test or one-way ANOVA, unless otherwise indicated. The association between EBV-miR-BART11-(3p and 5p) and *FOXP1* gene expression was analyzed using Spearman's correlation coefficient. *p* < 0.05 was considered statistically significant.

## SUPPLEMENTARY FIGURE AND TABLE




