# Supplementary material for: Epstein-Barr virus encoded miR-BART11 promotes inflammation-induced carcinogenesis by targeting FOXP1
Source: Oncotarget. 2016 May 4;7(24):36783–99. doi: 10.18632/oncotarget.9170 (PMC5095039; doi:10.18632/oncotarget.9170)
Supplement: Supplementary file 1 [file oncotarget-07-36783-s001.pdf]

SUPPLEMENTARY FIGURE AND TABLE

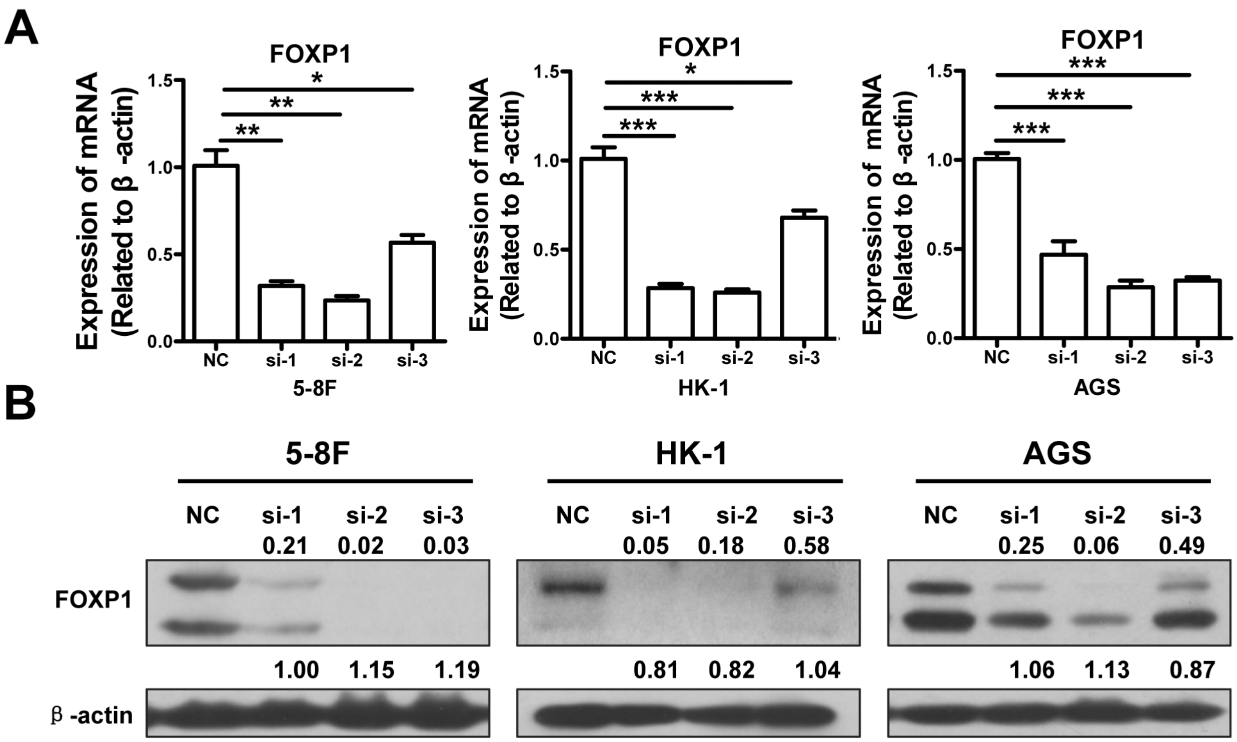

Supplementary Figure S1: The effects of siFOXP1 on the expression of FOXP1. A. The expression of FOXP1 in 5-8F, HK-1, and AGS cells transfected with three siRNAs that specifically targeted FOXP1 (siFOXP1). **B.** The expression of FOXP1 protein following the siFOXP1 transfection. The results are representative images or expressed as mean ± SD obtained using different cell lines in three independent experiments. (*$p < 0.05$; **$p < 0.01$; ***$p < 0.001$).

Supplementary Table S1: Primers used for qRT-PCR or construction and siRNA.

See Supplementary File 1
